# Brain Responses to High-Calorie Visual Food Cues in Individuals with Normal-Weight or Obesity: An Activation Likelihood Estimation Meta-Analysis

**DOI:** 10.3390/brainsci11121587

**Published:** 2021-11-30

**Authors:** Yingkai Yang, Qian Wu, Filip Morys

**Affiliations:** 1 Faculty of Psychology, Southwest University, No. 2 Tiansheng Street, Beibei District, Chongqing 400715, China; 2 The Lab of Mental Health and Social Adaptation, Faculty of Psychology, Research Center of Mental Health Education, Southwest University, Chongqing 400715, China; chelle@email.swu.edu.cn; 3 Montreal Neurological Institute, McGill University, Montreal, QC H3A 2B4, Canada; filip.morys@mail.mcgill.ca

**Keywords:** high-calorie food cues, neuroimaging, normal-weight, obesity, meta-analysis

## Abstract

Overconsumption of high-calorie or unhealthy foods commonly leads to weight gain. Understanding people’s neural responses to high-calorie food cues might help to develop better interventions for preventing or reducing overeating and weight gain. In this review, we conducted a coordinate-based meta-analysis of functional magnetic resonance imaging studies of viewing high-calorie food cues in both normal-weight people and people with obesity. Electronic databases were searched for relevant articles, retrieving 59 eligible studies containing 2410 unique participants. The results of an activation likelihood estimation indicate large clusters in a range of structures, including the orbitofrontal cortex (OFC), amygdala, insula/frontal operculum, culmen, as well as the middle occipital gyrus, lingual gyrus, and fusiform gyrus. Conjunction analysis suggested that both normal-weight people and people with obesity activated OFC, supporting that the two groups share common neural substrates of reward processing when viewing high-calorie food cues. The contrast analyses did not show significant activations when comparing obesity with normal-weight. Together, these results provide new important evidence for the neural mechanism underlying high-calorie food cues processing, and new insights into common and distinct brain activations of viewing high-calorie food cues between people with obesity and normal-weight people.

## 1. Introduction

The prevalence of obesity is problematic and rising in both developed and developing nations [1]. This fact has far-reaching and costly implications, because obesity contributes to the development of numerous diseases (e.g., diabetes, some cancers) [2,3,4], and it is a risk factor for psychiatric disorders (e.g., depression, anxiety) [5]. Not surprisingly, excessive weight has become an increasing threat to healthcare systems [6], and accounts for an estimated 2.8 million deaths per annum worldwide [7]. These statistics have prompted a plethora of research aimed at understanding factors that contribute to the development or maintenance of obesity [8,9,10,11,12].

One contributing factor is the overconsumption of high-calorie or unhealthy foods (e.g., chocolate cake), and underconsumption of low-calorie or healthy foods (e.g., salad), which leads to a positive energy balance and, subsequently, weight gain [13,14,15]. We are currently facing the rise of the ‘obesogenic’ environment [16] where the exposure to food advertisements, and availability of cheap, unhealthy, and energy dense foods has dramatically increased [17,18]. The constant exposure to high-calorie foods and food cues may promote overconsumption by stimulating brain reward and motivation pathways [19,20]. In this vein, using techniques such as functional magnetic resonance imaging (*f*MRI), a growing number of research has been conducted to investigate neural responses to various forms of food stimuli [21], such as liquid tastants, food odors [22], or visual food cues [23,24]. Moreover, recent reviews have used *f*MRI-based meta-analysis such as Activation Likelihood Estimation (ALE) [25,26] to evaluate the consistency of findings across these studies [23,24,27,28,29,30,31,32]. For instance, Chen and Zeffiro meta-analyzed 39 experiments with 995 participants and found that taste (e.g, insula), sensory integration (e.g., postcentral gyrus), and reward processing (e.g., amygdala) regions were involved in processing sweet food cues (one kind of high-calorie foods) [30]. With regard to visual food cues, several *f*MRI-based meta-analyses have also been conducted [18,24,27,28]. For example, an ALE meta-analysis including 12 experiments and 201 participants reported that visual food cues were reliably associated with increased blood oxygen level dependent (BOLD) response in the visual system proper (e.g., the occipital lobe) rather than reward-related brain network (e.g., the orbitofrontal cortex) [28].

None of the aforementioned meta-analyses, however, have investigated which brain regions are concurrently activated in response to viewing high-calorie food cues specifically. Furthermore, most of these meta-analyses only included participants with normal-weight and did not consider individuals with obesity (i.e., body mass index ≥30. A meta-analysis pooling data across relevant *f*MRI studies would therefore be warranted, as it may help to understand neural responses of viewing high-calorie food cues among people with various weight-status categories (e.g., normal-weight, obesity) and develop better interventions for preventing or reducing overeating and obesity.

In the present study, we conducted *f*MRI meta-analyses of viewing high-calorie food cues to address four novel aspects. First and foremost, we investigated brain activations related to high-calorie visual food cues. Second, we meta-analyzed not only studies involving normal-weight people, but also studies involving participants with obesity. Third, we performed conjunction and contrast analyses to assess common and distinct brain activations between normal-weight people and people with obesity (e.g., normal-weight ∩ obesity; normal-weight versus obesity). Last but not least, we utilized a new version of GingerALE brain map software (3.0.2) which was revised because of the implementation errors in multiple comparisons corrections in its old version [33].

## 2. Methods

### 2.1. Study Selection and Inclusion Criteria

**Figure 1:**
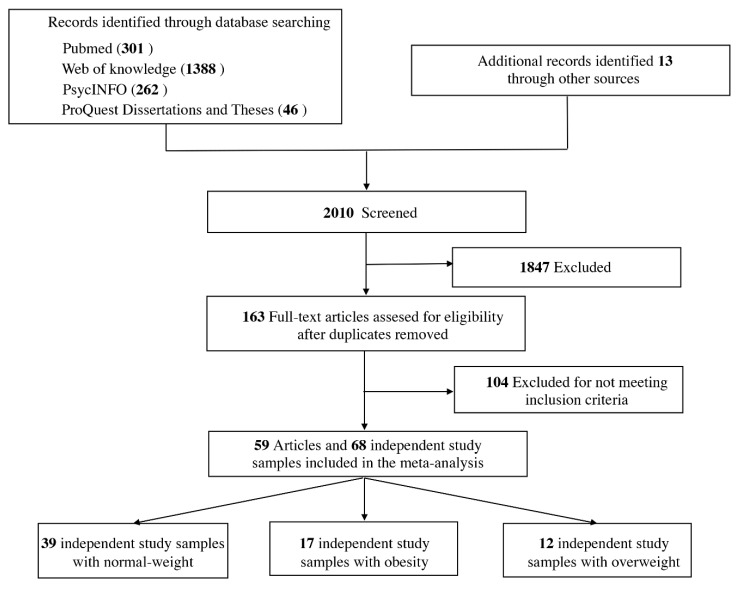
Flow diagram illustrating the process of our review, screening, and article selections.

The current meta-analysis was performed according to the Preferred Reporting Items for Systematic reviews and Meta Analyses guidelines (Supplementary Materials Table S1). A protocol for this work was registered on the Open Science Framework (OSF: https://osf.io/mvzn8). To obtain functional imaging studies of viewing high-calorie food cues for use in the current meta-analysis, a topic search in the databases PubMed, ISI Web of Knowledge, PsycINFO, and ProQuest Dissertations & Theses was conducted for all papers published before 9th August 2021. The following string was used to search for the titles, abstracts and keywords of papers: ((neuroimaging OR “functional magnetic resonance imaging” OR “positron-emission tomography” OR PET OR fMRI) AND (food) AND (images OR pictures)). In this search, PubMed returned 301 results, Web of Science returned 1388 results, PsycINFO returned 262 results, and ProQuest Dissertations & Theses returned 46 results. Abstracts of articles were reviewed, and the full text of an article was read whenever a paper’s title or abstract indicated that the study might be relevant to analyses. In addition, to help ensure that all studies on the topic of interest were included, references from relevant articles were reviewed, and studies that were potentially relevant were examined from those references. Figure 1 outlines the detailed study selection procedure.

Studies were incorporated into this research if they (1) were in English, (2) used *f*MRI, (3) used whole-brain analysis, (4) involved populations with normal-weight, overweight, or obesity. In samples of adult participants, a group with obesity was defined as an average body mass index (BMI) of 30 kg/m^2^ or above, a group with overweight as an average BMI between 25 and 29.99 kg/m^2^, and a group with normal-weight as an average BMI between 18.5 and 24.99 kg/m^2^. In samples of children/adolescents, obesity was defined as a BMI percentile of 95th or above, overweight as a BMI between the 85th and 94.99th percentile, and normal-weight as a BMI between the 5th and 84.99th percentile; if BMI percentile was not reported, international cutoff points of BMI were used for defining obesity and overweight [34], (5) used a viewing task (e.g., passive viewing task) in which cues of high-calorie foods were presented; Studies employing inhibitory control task, food choice task, or delay discounting task were excluded, (6) reported analyses for the contrast high-calorie foods > non-foods or low-calorie foods, (7) brain coordinates were available in Montreal Neurological Institute or Talairach space. If information crucial to our meta-analysis was not available in an article, we requested it from the corresponding authors. In addition, we excluded studies that explicitly indicated that all their participants are from special population (e.g., cancer survivors) or had a known eating disorder (e.g., binge eating disorder). Finally, when two studies used overlapping samples, we only included the study with a larger sample size.

### 2.2. Activation Likelihood Estimation Analysis

Meta-analyses were conducted in GingerALE brain map software (3.0.2) (www.brainmap.org), according to standard procedures outlined in the GingerALE user manual. Coordinates were extracted from whole-brain results, and those reported in Talairach space were transformed into Montreal Neurologic Institute (MNI) space using the automated transformation tool implemented in GingerALE. GingerALE algorithm minimizes within-experiment effects—clusters are more likely to represent agreement between experiments than being driven by individual research [35]. Because of this, we included all of the reported activation coordinates if a study included both the contrast high-calorie foods > non-food and high-calorie foods > low-calorie foods, as it was unlikely that a single study would bias the analyses (see Supplementary Materials Tables S3–S6 for the results of analyzing high-calorie vs. neutral and high-calorie vs. low-calorie contrasts separately). Similarly, foci obtained from contrasts in fasting and fed conditions were also merged.

As a first step, we performed an overall meta-analysis on activation foci derived from all the included articles investigating brain activation in response to viewing high-calorie visual food cues. We then performed two separate ALE analyses on two categories of studies in relation to the weight status of the participants (normal-weight or obesity) (Because only 12 independent samples included participants with overweight (for more, see Section 3.1), we do not present a separate meta-analysis on these overweight-related studies given the lack of statistical power). Following the guidelines of previous work [25,26,36], the cluster determining threshold was set at a cluster-level threshold of *p* < 0.01 and a voxel-level cluster forming threshold of *p* < 0.001 to correct for multiple comparisons.

### 2.3. Modulation Effect of Sex

We extracted per-voxel probabilities of activation in the meta-analysis for each of the identified brain regions to examine potential modulating effect of sex on the results of our analysis.

### 2.4. Conjunction and Contrast Analyses

To compare the results of pairwise meta-analysis (e.g., normal weight vs. obesity; normal weight vs. overweight/obesity), we performed conjunction and contrast analyses in GingerALE. Following the recommendations by Eickhoff et al. (2016) [36], the cluster determining threshold was set at *p* < 0.01 with 10,000 permutations and a minimum cluster size of 200 mm^3^.

### 2.5. Results Visualization

For visualization purposes, all results were projected onto an MNI-space template brain (e.g., MNI152.nii) using MRIcroGL (https://www.nitrc.org/projects/mricrogl/).

### 2.6. Study Quality Assessment

A 7-item quality scale modified from Nichol et al. (2018) [37] was used to assess the quality of the included studies (see Supplementary Materials Table S2 for study quality of each included study).

## 3. Results

### 3.1. Included Studies and Sample Characteristics

Our search identified 59 eligible studies (total *m* = 59) [38,39,40,41,42,43,44,45,46,47,48,49,50,51,52,53,54,55,56,57,58,59,60,61,62,63,64,65,66,67,68,69,70,71,72,73,74,75,76,77,78,79,80,81,82,83,84,85,86,87,88,89,90,91,92,93,94,95,96], 68 independent samples (total *k* = 68), and a total of 2410 participants (total *N* = 2410). A complete list of studies and their characteristics can be seen in Table 1. Of these 59 studies, 39 independent samples (*k* = 39, *N* = 979) included participants with normal-weight, 17 independent samples (*k* = 17, *N* = 697) included participants with obesity, and 12 independent samples (*k* = 12, *N* = 734) included participants with overweight.

### 3.2. Overall Meta-Analysis

**Figure 2:**
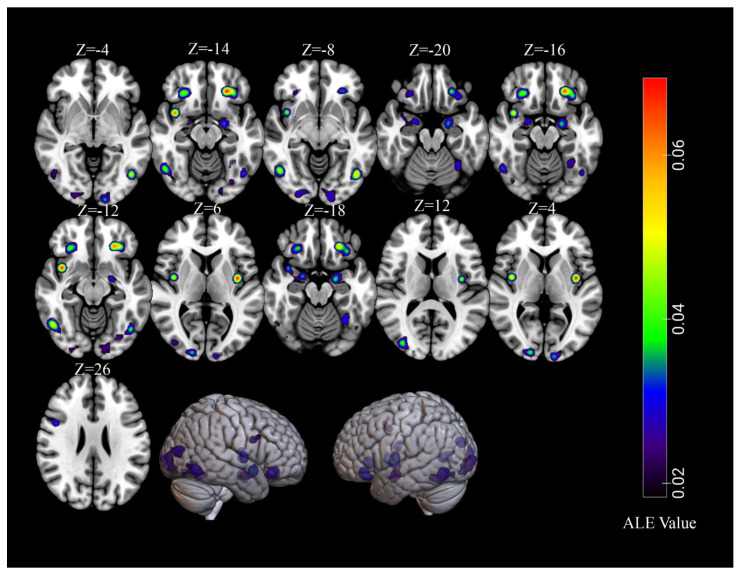
Significant clusters from the overall meta-analysis for viewing high-calorie food cues.

The primary meta-analysis that pooled data across all 68 independent samples (854 foci) revealed that high-calorie food cues activated thirteen statistically significant clusters (total volume of activation of 33,632 mm^3^ and maximum ALE value of 0.0694). The results are reported in Table 2 and graphically represented in Figure 2. We observed that viewing high-calorie food cues consistently activated the bilateral lingual gyrus, fusiform gyrus, orbitofrontal cortex (OFC), amygdala, insula, as well as the right middle occipital gyrus, left culmen, and the right inferior frontal gyrus (IFG). For all of the identified clusters, the modulation analysis revealed no effect of sex (all *p* values > 0.05).

### 3.3. Brain Response to High-Calorie Visual Food Cues in People with Normal-Weight

**Figure 3:**
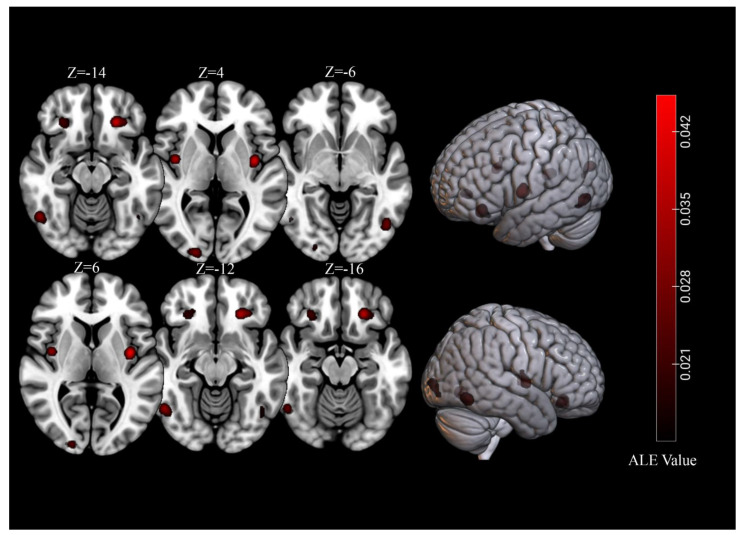
Significant clusters for of viewing high-calorie food cues in samples of individuals with normal-weight.

For brain activations of viewing high-calorie food cues in participants with normal-weight, the meta-analysis of 39 independent samples (493 foci) identified seven significant clusters (total volume of activation of 10,680 mm^3^ and maximum ALE value of 0.0713) that covered regions of the bilateral fusiform gyrus, OFC, insula, as well as the right lingual gyrus (Table 3, Figure 3).

### 3.4. Brain Response to High-Calorie Visual Food Cues in People with Obesity

**Figure 4:**
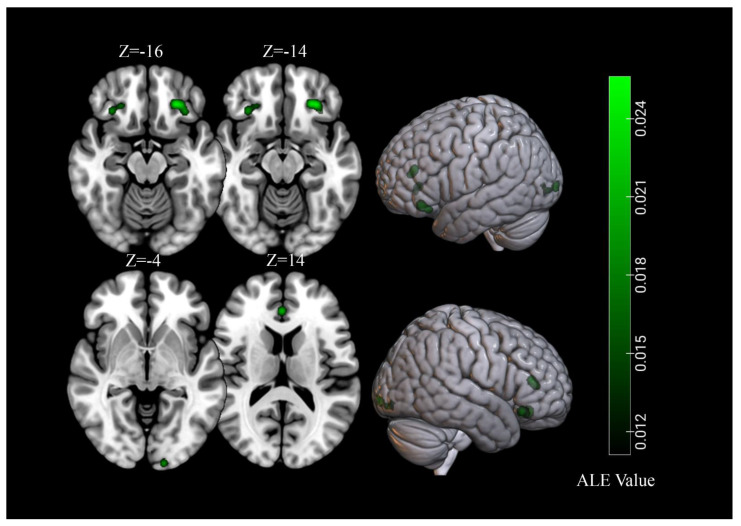
Significant clusters for viewing high-calorie food cues in samples of individuals with obesity.

For brain activations of viewing high-calorie food cues in participants with obesity, the meta-analysis of 17 independent samples (216 foci) identified seven significant clusters (total volume of activation of 4952 mm^3^ and maximum ALE value of 0.0533) that covered regions of the bilateral OFC, left lingual gyrus, and the anterior cingulate cortex (Table 3, Figure 4).

### 3.5. Conjunction and Contrast Analyses

**Figure 5:**
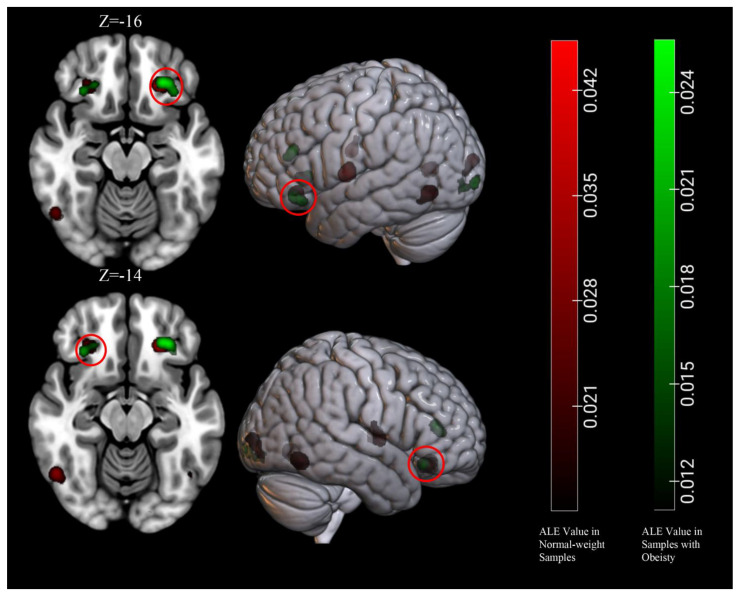
Significant common clusters of viewing high-calorie food cues across samples of individuals with normal-weight and obesity.

To identify clusters of activation which were common to normal-weight people and people with obesity, we carried out a conjunction analysis on the activations obtained in the previous two separate meta-analyses. The results revealed significant activations in the bilateral OFC, which was commonly activated in both normal-weight people and individuals with obesity (Table 4, Figure 5).

To identify clusters of activation which were unique to normal-weight people and people with obesity, a contrast analysis was carried out between the activations obtained in the previous two separate meta-analyses. The analysis revealed no significant activations.

Finally, we conducted additional analyses with overweight and obese as one group and compared this combined group with the normal weight group (Table 4). Conjunction analysis revealed significant activations in the bilateral OFC, fusiform gyrus, and the left insula, which were commonly activated in both normal-weight people and individuals with obesity/overweight. The obesity/overweight minus normal-weight subtraction analysis displayed 2 significant clusters located in the bilateral culmen.

## 4. Discussion

By meta-analyzing 59 *f*MRI studies and 68 independent samples, we showed a network of brain regions related to viewing high-calorie food cues. Further, we presented two separate meta-analyses to examine neural responses of viewing high-calorie food cues in normal-weight people or people with obesity, and investigated the common and differential neural responses between these two groups. Although some meta-analyses have been conducted on this topic before [23,24,27,28], to the best of our knowledge, we are the first group to examine the neural mechanisms of viewing high-calorie food cues across groups with a different weight-status.

We found that, overall, viewing high-calorie food cues was associated with increased activity in a network of brain regions located in the bilateral lingual gyrus, fusiform gyrus, OFC, amygdala, insula, as well as the right middle occipital gyrus, left culmen, and the right IFG. The conjunction analysis suggested that viewing high-calorie food cues activated the OFC in both normal-weight people and people with obesity. Different from the conclusions from previous reviews on the topic of food cue reactivity in obesity [97,98], but similar to the viewpoint of a behavioral meta-analysis of food cue reactivity [11], the contrast analysis revealed no significant activations when comparing groups with obesity to groups with normal-weight (more on this in Section 4.2).

### 4.1. Core Brain Regions Activated by High-Calorie Visual Food Cues

Our overall results are similar to a previous meta-analysis focusing on the functional neuroanatomy of high-calorie food liquid processing (e.g., sweet liquid) [30]. 

The amygdala and OFC are connected with each other and frequently activated in food studies. The amygdala is thought to form the core of a neural system for fear processing [99]. However, accumulating evidence indicates that the amygdala also plays a prominent role in mediating positive/reward stimuli processing [100]. These findings have led to the viewpoint that the amygdala’s predominant role may be the detection of and response to motivationally important stimuli [101]. In addition, it was proposed that the amygdala was responsible for forming an “affective tag” to the salient stimuli [102]. Therefore, the amygdala activations that we found in current meta-analysis are likely to reflect the salience and emotional impacts of high-calorie food cues. The OFC receives information from brain regions involved in sensory processing (e.g., insula, fusiform gyrus), affective processing (e.g., amygdala), and memory (e.g., hippocampus), and plays a prominent role in integrating, encoding, and retrieving reward value about stimulus [103]. There is a strong and consistent activation of the OFC in reward-related tasks such as decision-making tasks [104] or cue-reactivity tasks [105]. Further, several studies have shown that the magnitude of activity in this region correlated with pleasantness or tastiness ratings of food/food cues [106,107,108]. Therefore, the OFC activations in current study could reflect the process of monitoring and encoding higher reward value of high-calorie food cues. It should be noted that we did not find that “classical” reward areas such as the nucleus accumbens, putamen, or caudate were involved in processing of high-calorie visual food cues. This is different from a previous meta-analysis which showed that the putamen and caudate exhibited responses to high-calorie liquids [30]. Although comparing across meta-analyses is difficult given differences in included studies, we could speculate that reward areas are more likely to be activated when people are eating/tasting high-calorie foods rather than viewing high-calorie food cues.

The insula/frontal operculum has been identified as the primary taste cortex [109,110]. The activation of this taste cortex in response to high-calorie visual food cues may represent memory retrieval of previous gustatory experiences with these palatable foods [23]. In addition, the insula has also been highlighted as a region that plays an important role in craving for drugs (e.g., cocaine) [111] and foods [112]. Therefore, it is also possible that insula activation is the result of high urges to eat in participants exposed to calorie-rich and appetizing food pictures [27].

We also found some evidence that the culmen was activated by high-calorie food cues. Although traditionally considered a major motor structure of the brain, there is evidence that the culmen/cerebellum plays a broader role in homeostatic regulation [113], and shows connections with limbic and reward systems [114]. Given that both the meta-analysis of neural responses to sweet stimuli by Chen and Zeffiro [30] and the current meta-analysis found that the cerebellum increases activity in response to food simulation, future studies and theories of eating behavior may benefit from inclusion of cerebellar influences in their hypotheses forming process.

The remiaining significant clusters found in response to high-calorie visual food cues were located in the occipitotemporal gyrus (the bilateral lingual gyrus and fusiform gyrus, the right middle occipital gyrus). These visual areas consistently respond to multiple drug-related (e.g., alcohol, cocaine, marijuana, tobacco) [115] and gaming cues [116]. Drawing parallels it is conceivable that higher reward salience of higher reward salience of high-calorie food images modulate neural activity in these visual areas, just as drug-related cues do, and leads to different visual processing when compared to control images.

### 4.2. Common and Specific Brain Activations between Normal-Weight and Obesity

From an evolutionary perspective, energy-dense foods confer a greater survival advantage for primate species, including humans. From this viewpoint, researchers argued that our species has a natural preference for high-calorie foods [117], which then could be perceived as more rewarding. In light of this, the results of our conjunction analysis–increased activity in the OFC, a reward-related brain area – are not surprising.

The null findings in the contrast analysis between groups of individuals with normal-weight and obesity are different from the conclusions from previous reviews on the topic of cue reactivity in obesity. Past work showed that obesity was related to an enhanced reward/salience response towards (high-calorie) food stimuli [97,98]. It should be noted that these reviews included studies using other stimuli than high-calorie food pictures (e.g., gustatory stimuli, such as chocolate milkshakes), which might lead to different conclusions than our work. Indeed, when only included studies using food pictures, two newly published meta-analyses found similar results to current work [118,119]. For example, Morys and colleagues [118] meta-analyzed 13 studies that investigated group differences (obese vs. normal-weight) in responses to food vs. non-food pictures viewing, and found little evidence for obesity-related differences in brain responses to food cues. In addition, our results are in line with the behavioral literature, as a meta-analysis including 45 published reports did not find evidence for the influence of BMI on food cue reactivity [11]. Taken together, current evidence tends to support that there are no (high-calorie) visual food cue reactivity differences between normal-weight people and people with obesity. Intuitively, this conclusion might contradict some findings of longitudinal studies on the topic of cue reactivity, which found that behavioral and neural responses to food cues predict weight gain [11,120]. However, researchers argued that food cue reactivity is not the only factor that influences food intake and weight gain [9,12,97,118]. For instance, theories proposed that reactions to hyper-palatable food cues might lead to increased food intake and weight gain only in individuals with lower dietary self-regulation, though future research should examine whether this is the case.

### 4.3. Limitations and Future Directions

Despite its strengths, our meta-analysis has some limitations. First and foremost, like most *f*MRI meta-analyses, we incorporated only the reported significant brain activation peaks from the included studies, which resulted in some information loss (e.g., not significant small clusters of activations) of the original fMRI data. We recommend that future studies share their original data or unthresholded statistical maps on a data repository such as Neurovault (e.g., https://neurovault.org), and an image-based meta-analysis of the neural correlates of viewing high-calorie food cues will be possible when sufficient raw data or statistical maps are available. Second, not all corresponding authors answered our requests for data. In addition, some studies which would be eligible for our meta-analysis did not report the main effects of high-calorie visual food cues on BOLD changes. As such, we recommend that future studies report these results in their Supplementary Materials  even if these contrasts might not be the key focus of the study. Next, obesity was operationalized via BMI in this meta-analysis, which is a relatively coarse measure of body density and may overlook relevant physical characteristics, such as body fat and anthropometric features. Last but not least, we did not examine the neural effect of high-calorie visual food cues in people who are overweight because of the relatively small number of studies. Moreover, compared to the number of normal-weight studies, the number of studies involved people with obesity was relatively small. Thus, more research is needed to investigate the neural effects of viewing high-calorie food cues in people with overweight/obesity.

## 5. Conclusions

In conclusion, the results of our meta-analysis suggest a core neural network of viewing high-calorie food cues, which comprise reward-related as well as visual brain areas and brain regions related to taste processing. The conjunction analysis suggests that groups of individuals with normal-weight and obesity share common neural substrates of reward processing when viewing high-calorie food cues. Finally, there seem to be no differences in neural processing of high-calorie food images between people with normal-weight and obesity. Together, our work provides the first meta-analytic evidence for the neural mechanism underlying high-calorie food cues processing and new insights into common and distinct brain activations of viewing high-calorie food cues.

## Figures and Tables

**Table 1:** Details of the 59 analyzed studies.

Study	N (Percent Female)	Mean Age	Weight Status	Hours Fasted	High-Calorie Food Cues	Control Stimuli	Task	Foci	*p*
Basso et al., 2018 [38]	20 (50%)	26	Normal-weight	At least 4	Sweet and salty food images	Non-food control images/Healthy food images	Passive viewing	16	*p* < 0.05, FWE corrected
Basu et al., 2016 [39]	8 (100%)	23	Normal-weight	At least 8	High-calorie food images	Low-calorie food images	Passive viewing	7	*p* < 0.05, corrected
Beaver et al., 2006 [40]	12 (58%)	22	Normal-weight	At least 2	Highly appetizing food images such as chocolate, ice cream	Non-food control pictures/Bland food images	Passive viewing	32	*p* < 0.001, uncorrected
Blechert et al., 2016 [41]	32 (50%)	22	Normal-weight	At least 3	Sweet and salty snack food images	Fruit, vegetables images	Passive viewing	25	*p* < 0.005, uncorrected
Carnell et al., 2017 [42]	10 (70%)/16 (50%)/10 (50%)	16	Normal-weight/Obesity	At least 5	High-calorie food words	Non-food words/Low-calorie food words	Passive viewing	21	*p* < 0.000005, uncorrected
Chen et al., 2017 [43]	36 (100%)	20	Normal-weight	N.A	Appetizing food images	Non-food control images	Viewing, attentional task	11	*p* < 0.05, corrected
Cornier et al., 2007 [47]	25 (50%)	35	Normal-weight	At least 10	High hedonic value food images	Neutral hedonic food images	Passive viewing	7	*p* < 0.05, FDR corrected
Cornier et al., 2009 [46]	22 (45%)	34	Normal-weight	At least 10	High hedonic value food images	Non-food control images	Passive viewing	23	*p* < 0.05, FDR corrected
Cornier et al., 2012 [45]	12 (42%)	38	Obesity	At least 10	High hedonic value food images	Non-food control images	Passive viewing	8	*p* < 0.01, FDR corrected
Cornier et al., 2013 [44]	25 (44%)/28 (50%)	31/30	Normal-weight/Overweight	At least 10	High hedonic value food images	Non-food control images	Passive viewing	6/9	*p* < 0.05, FDR corrected
Davids et al., 2010 [48]	22 (45%)/22 (32%)	14/14	Normal-weight/Obesity	At least 2	Pizza, hamburgers, sweets images	Non-food control images	Passive viewing	13/13	*p* < 0.05, FDR corrected
Doornweerd et al., 2018 [49]	32 (100%)	50	Overweight	At least 12	High-calorie food images	Non-food control images	Passive viewing	5	*p* < 0.05, FWE corrected
English et al., 2017 [50]	36 (50%)	9	Normal-weight	At least 2	High-energy food images	Low-energy food images	Passive viewing	10	*p* < 0.05, corrected
Evero et al., 2012 [51]	30 (43%)	22	Normal-weight	At least 10	High-energy food images	Non-food control images	Passive viewing	1	*p* < 0.005, uncorrected
Frank et al., 2010 [52]	12 (50%)	27	Normal-weight	Fast/fed	High-calorie food images	Non-food control images/Low-calorie food images	Viewing, attentional task	21	*p* < 0.05, FDR corrected
Frank et al., 2014 [53]	31 (100%)	41	Obesity	0.5	High-calorie food images	Non-food control images/Low-calorie food images	Viewing, attentional task	22	*p* < 0.001, uncorrected
García-García et al., 2020 [54]	58 (100%)	26	Overweight	At least 2	Palatable food images	Non-food control images	Passive viewing	7	*p* < 0.05, FWE corrected
Gearhardt et al., 2020 [55]	171 (51%)	14	Overweight	At least 3	High-calorie food commercials	Non-food commercials/Low-calorie food commercials	Passive viewing	45	*p* < 0.05, corrected
Geliebter et al., 2013 [56]	31 (45%)	35	Obesity	Fast/fed	High-energy food images	Low-energy food images	Passive viewing	16	*p* < 0.005, uncorrected
Goldstone et al., 2009 [57]	20 (50%)	26	Normal-weight	Fast/fed	High-energy food images	Low-energy food images	Passive viewing	42	*p* < 0.05, FDR corrected
Heni et al., 2014 [58]	24 (50%)	24	Overweight	At least 10	High-calorie food images	Low-calorie food images	Passive viewing	7	*p* < 0.001, uncorrected
Hermann et al., 2019 [59]	29 (90%)	48	Obesity	At least 2	Sweet and salty snack images	Low-calorie food images	Passive viewing	13	*p* < 0.05, FDR corrected
Horster et al., 2020 [60]	27 (89%)	24	Normal-weight	N.A	Sweet and savoury food images	Non-food control images	Passive viewing	6	*p* < 0.05, FWE corrected
Jastreboff et al., 2013 [62]	25 (40%)	26	Obesity	2	High-calorie food images	Neutral-relaxing images	Passive viewing	6	*p* < 0.01, FWE corrected
Jastreboff et al., 2014 [61]	25 (60%)/15 (33%)	16	Normal-weight/Obesity	2	High-calorie food images	Non-food control images/Low-calorie food images	Passive viewing	8/4	*p* < 0.01, FWE corrected
Jensen & Kirwan, 2015 [63]	34 (85%)	19	Overweight	At least 4	High-energy food images	Low-energy food images	Passive viewing	7	*p* < 0.05, corrected
Karra et al., 2013 [64]	24 (0%)	23	Normal-weight	Fast/fed	High-calorie food images	Low-calorie food images	Passive viewing	5	*p* < 0.001, uncorrected
Killgore et al. 2003 [66]	13 (100%)	24	Normal-weight	6	High-calorie food images	Non-food control images	Passive viewing	18	*p* < 0.0005, uncorrected
Killgore et al. 2005 [65]	8 (100%)	12	Normal-weight	6	High-calorie food images	Non-food control images/Low-calorie food images	Passive viewing	17	*p* < 0.005, uncorrected
Kim et al., 2012 [67]	20 (100%)	23	Normal-weight	6	High-calorie food images	Non-food control images	Passive viewing	4	*p* < 0.001, uncorrected
Le et al., 2021 [68]	82 (40%)	41	Overweight	4	High-calorie food images	Non-food control images	Passive viewing	18	*p* < 0.05, FWE corrected
Li et al., 2021 [69]	118 (58%)	27	Obesity	At least 12	High-calorie food images	Low-calorie food images	Passive viewing	3	*p* < 0.05, FWE corrected
Luo et al., 2013 [71]	13 (100%)	23	Obesity	At least 10	High-calorie food images	Non-food control images	Passive viewing	18	*p* < 0.05, FWE corrected
Luo et al., 2019 [70]	53 (58%)	8	Normal-weight	At least 12	High-calorie food images	Non-food control images	Passive viewing	29	*p* < 0.05, FWE corrected
Malik et al., 2011 [72]	10 (0%)	26	Normal-weight	At least 8	High-calorie food images	Non-food control images	Passive viewing	27	*p* < 0.05, corrected
Masterson et al., 2016 [73]	15 (100%)	23	Normal-weight	At least 6	High-calorie food images	Low-calorie food images	Viewing, attentional task	9	*p* < 0.001, uncorrected
Mengotti et al., 2016 [74]	25 (56%)	24	Normal-weight	At least 4	High-calorie food images	Low-calorie food images	Viewing, attentional task	6	*p* < 0.001, uncorrected
Merchant et al., 2020 [75]	93 (83%)	39	Obesity	At least 1	High-caloric snack food images	Low-calorie food images	Passive viewing	6	*p* < 0.05, FWE corrected
Murdaugh et al., 2012 [76]	25(76%)/13(76%)	48/45	Normal-weight/Obesity	At least 8	Sweet foods images	Non-food control images	Passive viewing	15/11	*p* < 0.05, FDR corrected
Murray et al., 2014 [77]	20 (50%)	23	Normal-weight	At least 2	Chocolate images	Grey images	Passive viewing	9	*p* < 0.05, FWE corrected
Neseliler et al., 2017 [78]	22 (59%)	21	Normal-weight	At least 4	High-calorie food images	Low-calorie food images	Passive viewing	4	*p* < 0.05, corrected
Nummenmaa et al., 2012 [79]	35 (50%)	47	Obesity	At least 3	Highly appetizing food images such as chocolate, pizza, steak	Low-calorie food images	Passive viewing	20	*p* < 0.05, FDR corrected
Passamonti et al., 2009 [80]	21 (48%)	25	Normal-weight	At least 2	High-calorie food images	Low-calorie food images	Passive viewing	13	*p* < 0.001, uncorrected
Pursey et al., 2019 [81]	11 (100%)	24	Overweight	Fast/fed	High-calorie food images	Low-calorie food images	Passive viewing	6	*p* < 0.001, uncorrected
Rapuano et al., 2016 [82]	37 (54%)	14	Overweight	At least 2	High-calorie food commercials	Non-food commercials	Passive viewing	5	*p* < 0.05, FWE corrected
Rothemund et al., 2007 [83]	13 (100%)	31	Obesity	At least 1.5	High-calorie food images	Non-food control images	Passive viewing	7	*p* < 0.05, FWE corrected
Santel et al., 2006 [84]	10 (100%)	17	Normal-weight	At least 12	Sweet and salty food images	Non-food control images	Passive viewing	7	*p* < 0.001, uncorrected
Schienle et al., 2009 [85]	19 (100%)/17 (100%)	22/25	Normal-weight/Obesity	At least 10	High-calorie food images	Low-calorie food images	Passive viewing	3/1	*p* < 0.05, FWE corrected
Simmons et al., 2005 [86]	9 (67%)	18–45	Normal-weight	N.A	Sweet and salty food images	Non-food control images	Passive viewing	6	*p* < 0.005, uncorrected
Smeets et al., 2013 [87]	30 (100%)	22	Normal-weight	3	Fattening food images	Non-food control images	Passive viewing	25	*p* < 0.001, uncorrected
St-Onge et al., 2014 [88]	25 (50%)	35	Normal-weight	At least 10	Unhealthy food images	Healthy food images	Passive viewing	20	*p* < 0.05, uncorrected
van Bloemendaal et al., 2014 [89]	48 (50%)	58	Obesity	N.A	High-calorie food images	Non-food control images	Passive viewing	20	*p* < 0.05, FWE corrected
van Meer et al., 2016 [90]	27 (67%)/32 (67%)	11/44	Normal-weight/Overweight	At least 2	Unhealthy food images	Healthy food images	Passive viewing	6/3	*p* < 0.05, corrected
van Meer, 2017 [95]	168 (56%)/183 (52%)	13/45	Normal-weight/Overweight	At least 2	High-calorie food images	Low-calorie food images	Passive viewing	11/26	*p* < 0.05, FWE corrected
Wabnegger et al., 2018 [91]	25 (100%)	24	Normal-weight	At least 10	High-caloric sweet foods images	Low-calorie food images	Passive viewing	4	*p* < 0.05, FWE corrected
Wagner et al., 2012 [92]	30 (100%)	20	Normal-weight	N.A	High-calorie food images	Non-food control images	Viewing, attentional task	10	*p* < 0.05, FWE corrected
Wang et al., 2016 [93]	24 (100%)	22	Normal-weight	4	High-energy food images	Non-food control images/Low-calorie food images	Passive viewing	8	*p* < 0.05, FDR corrected
Yang et al., 2021 (unpublished data) [96]	42 (93%)	19	Overweight	2	High-calorie food images	Low-calorie food images	Passive viewing	7	*p* < 0.05, FWE corrected
Yokum et al., 2021 [94]	150 (79%)	30	Obesity	3	High-calorie food images	Glass of water images/Low-calorie food images	Passive viewing	36	*p* < 0.05, corrected

Note: N.A = Not available; N = Sample size; FEW = Family-Wise Error; FDR = False Discovery Rate.

**Table 2:** Overall Activation Likelihood Estimation meta-analysis of high-calorie visual food stimuli relative to a control condition using 68 independent samples (59 studies).

Cluster	Cluster Size (mm^3^)	Brain Region	Peak Voxel MNI Coordinates			ALE Value (×10^−2^)	Z	Contributing Samples	
			X	Y	Z			No.	%
1	4096	L Lingual Gyrus	−14	−98	−4	3.65	5.28	20	29%
2	3680	L Orbitofrontal Cortex	−26	34	−14	6.94	8.40	21	31%
3	3368	R Lingual Gyrus	22	−90	−8	2.88	4.43	18	26%
4	3232	R Amygdala	28	−6	−20	2.30	3.71	17	25%
5	3136	R Fusiform Gyrus	38	−76	−16	2.29	3.69	16	24%
6	3040	L Fusiform Gyrus	−30	−78	−12	2.63	4.13	18	26%
7	2512	R Orbitofrontal Cortex	26	32	−14	4.35	6.01	15	22%
8	2312	L Insula	−38	−6	6	6.90	8.36	16	24%
9	2184	L Amygdala	−20	−6	−18	3.94	5.59	13	19%
10	2168	R Middle Occipital Gyrus	36	−84	12	4.31	5.98	11	16%
11	1376	L Culmen	−32	−56	−18	3.27	4.87	7	10%
12	1352	R Insula	40	−4	4	5.46	7.09	10	15%
13	1176	R Inferior Frontal Gyrus	46	6	26	3.41	5.03	6	9%

Note: L: left, R: right. The presented clusters were significant at a *p* < 0.001 corrected for multiple comparisons using cluster-level family-wise error correction at a *p* < 0.01 (1000 permutations).

**Table 3:** Separate meta-analytic results of significant clusters in individuals with normal-weight or obesity.

Cluster	Cluster Size (mm^3^)	Brain Region	Peak Voxel MNI Coordinates			ALE Value (×10^−2^)	Z	Contributing Samples	
			X	Y	Z			No.	%
			Normal weight						
1	2080	L Orbitofrontal Cortex	−24	32	−14	4.01	6.56	9	23%
2	1600	R Lingual Gyrus	20	−96	4	2.92	5.36	8	21%
3	1568	L Fusiform Gyrus	−46	−68	−6	2.73	5.02	8	21%
4	1568	L Insula	−38	−6	6	4.53	7.13	9	23%
5	1560	R Fusiform Gyrus	50	−60	−12	3.23	5.65	7	18%
6	1160	R Insula	40	−4	4	3.62	6.11	8	21%
7	1144	R Orbitofrontal Cortex	28	32	−16	2.24	4.37	8	21%
				Obesity					
1	1680	L Orbitofrontal Cortex	−26	34	−16	2.56	5.33	6	35%
2	1344	L Lingual Gyrus	−16	−100	−4	1.96	4.47	6	35%
3	1000	R Orbitofrontal Cortex	32	28	−14	1.96	4.48	4	24%
4	928	Anterior Cingulate Cortex	0	36	14	2.15	4.75	5	29%

Note: L: left, R: right. These presented clusters were significant at a *p* < 0.001 corrected for multiple comparisons using cluster-level fami-ly-wise error correction at a *p* < 0.01 (1000 permutations).

**Table 4:** Conjunction and contrast analyses between samples with obesity/overweight and normal-weight.

Cluster	Cluster Size (mm^3^)	Brain Region	Peak Voxel MNI Coordinates			ALE Value (×10^−2^)/Z
			X	Y	Z	
Obesity ∩ Normal-weight	1232	L Orbitofrontal Cortex	−26	34	−16	2.56
	544	R Orbitofrontal Cortex	30	30	−14	1.80
Obesity > Normal-weight	None					
Obesity < Normal-weight	None					
Obesity/overweight ∩ Normal-weight	1344	L Orbitofrontal Cortex	−26	34	−14	3.53
	904	L insula	−38	−6	2	3.06
	864	L Fusiform Gyrus	−46	−68	−6	2.73
	784	R Fusiform Gyrus	48	−64	−10	2.61
	712	R Orbitofrontal Cortex	28	32	−14	2.15
Obesity/overweight > Normal-weight	584	L Culmen	27	−53.8	−13.7	3.19
	208	R Culmen	−26	−58	−16	2.66
Obesity/overweight < Normal-weight	None					

Note: L: left, R: right. The presented clusters were significant at a *p* < 0.01 with 10,000 permutations and a minimum cluster size of 200 mm^3^.

## Data Availability

Datasets arising from the study might be available upon reasonable request from the corresponding author.

## Supplementary Materials

The following are available online at https://www.mdpi.com/article/10.3390/brainsci11121587/s1, Tables S1–S6: Table S1. PRISMA Checklist; Table S2. Quality assessment of each included study; Table S3. Overall Activation Likelihood Estimation meta-analysis of high-calorie visual food stimuli relative to the non-food condition using 43 independent samples; Table S4. Conjunction and contrast analyses on activations of high-calorie visual food stimuli relative to the non-food condition between obesity and normal-weight; Table S5. Overall Activation Likelihood Estimation meta-analysis of high-calorie visual food stimuli relative to the low-calorie visual food condition using 39 independent samples; Table S6. Conjunction and contrast analyses on activations of high-calorie visual food stimuli relative to the low-calorie visual food condition between obesity and normal-weight.

## References

[1] Ng M., Fleming T., Robinson M., Thomson B., Graetz N., Margono C., Mullany E.C., Biryukov S., Abbafati C., Abera S.F. (2014). Global, regional, and national prevalence of overweight and obesity in children and adults during 1980-2013: A systematic analysis for the Global Burden of Disease Study 2013. Lancet.

[2] Lavie C.J., Milani R.V., Ventura H.O. (2009). Obesity and cardiovascular disease: Risk factor, paradox, and impact of weight loss. J. Am. Coll. Cardiol..

[3] Mokdad A.H., Ford E.S., Bowman B.A., Dietz W.H., Vinicor F., Bales V.S., Marks J.S. (2003). Prevalence of obesity, diabetes, and obesity-related health risk factors, 2001. JAMA.

[4] Kyrgiou M., Kalliala I., Markozannes G., Gunter M.J., Paraskevaidis E., Gabra H., Martin-Hirsch P., Tsilidis K.K. (2017). Adiposity and cancer at major anatomical sites: Umbrella review of the literature. BMJ.

[5] Luppino F.S., de Wit L.M., Bouvy P.F., Stijnen T., Cuijpers P., Penninx B.W., Zitman F.G. (2010). Overweight, obesity, and depression: A systematic review and meta-analysis of longitudinal studies. Arch. Gen. Psychiatry.

[6] Withrow D., Alter D.A. (2011). The economic burden of obesity worldwide: A systematic review of the direct costs of obesity. Obes. Rev..

[7] World Health Organization. https://www.who.int/news-room/facts-in-pictures/detail/6-facts-on-obesity.

[8] Vainik U., Dagher A., Realo A., Colodro-Conde L., Mortensen E.L., Jang K., Juko A., Kandler C., Sorensen T.I.A., Mottus R. (2019). Personality-obesity associations are driven by narrow traits: A meta-analysis. Obes. Rev..

[9] Yang Y., Shields G.S., Guo C., Liu Y. (2018). Executive function performance in obesity and overweight individuals: A meta-analysis and review. Neurosci. Biobehav. Rev..

[10] Yang Y., Shields G.S., Wu Q., Liu Y., Chen H., Guo C. (2020). The association between obesity and lower working memory is mediated by inflammation: Findings from a nationally representative dataset of U.S. adults. Brain Behav. Immun..

[11] Boswell R.G., Kober H. (2016). Food cue reactivity and craving predict eating and weight gain: A meta-analytic review. Obes. Rev..

[12] Lowe C.J., Reichelt A.C., Hall P.A. (2019). The Prefrontal Cortex and Obesity: A Health Neuroscience Perspective. Trends Cogn. Sci..

[13] Hill J.O. (2006). Understanding and addressing the epidemic of obesity: An energy balance perspective. Endocr. Rev..

[14] Hu F.B. (2013). Resolved: There is sufficient scientific evidence that decreasing sugar-sweetened beverage consumption will reduce the prevalence of obesity and obesity-related diseases. Obes. Rev..

[15] Ruanpeng D., Thongprayoon C., Cheungpasitporn W., Harindhanavudhi T. (2017). Sugar and artificially sweetened beverages linked to obesity: A systematic review and meta-analysis. QJM.

[16] Kirk S.F., Penney T.L., McHugh T.L. (2010). Characterizing the obesogenic environment: The state of the evidence with directions for future research. Obes. Rev..

[17] Gearhardt A.N., Bragg M.A., Pearl R.L., Schvey N.A., Roberto C.A., Brownell K.D. (2012). Obesity and public policy. Annu. Rev. Clin. Psychol..

[18] Swinburn B.A., Sacks G., Hall K.D., McPherson K., Finegood D.T., Moodie M.L., Gortmaker S.L. (2011). The global obesity pandemic: Shaped by global drivers and local environments. Lancet.

[19] Stice E., Burger K. (2019). Neural vulnerability factors for obesity. Clin. Psychol. Rev..

[20] Berridge K.C., Ho C.Y., Richard J.M., DiFeliceantonio A.G. (2010). The tempted brain eats: Pleasure and desire circuits in obesity and eating disorders. Brain Res..

[21] Yeung A.W.K., Wong N.S.M., Lau H., Eickhoff S.B. (2019). Human brain responses to gustatory and food stimuli: A meta-evaluation of neuroimaging meta-analyses. Neuroimage.

[22] Han P. (2021). Advances in research on brain processing of food odors using different neuroimaging techniques. Curr. Opin. Food Sci..

[23] Van der Laan L.N., de Ridder D.T., Viergever M.A., Smeets P.A. (2011). The first taste is always with the eyes: A meta-analysis on the neural correlates of processing visual food cues. Neuroimage.

[24] Van Meer F., van der Laan L.N., Adan R.A., Viergever M.A., Smeets P.A. (2015). What you see is what you eat: An ALE meta-analysis of the neural correlates of food viewing in children and adolescents. Neuroimage.

[25] Eickhoff S.B., Bzdok D., Laird A.R., Kurth F., Fox P.T. (2012). Activation likelihood estimation meta-analysis revisited. Neuroimage.

[26] Eickhoff S.B., Laird A.R., Grefkes C., Wang L.E., Zilles K., Fox P.T. (2009). Coordinate-based activation likelihood estimation meta-analysis of neuroimaging data: A random-effects approach based on empirical estimates of spatial uncertainty. Hum. Brain Mapp..

[27] Tang D.W., Fellows L.K., Small D.M., Dagher A. (2012). Food and drug cues activate similar brain regions: A meta-analysis of functional MRI studies. Physiol. Behav..

[28] Huerta C.I., Sarkar P.R., Duong T.Q., Laird A.R., Fox P.T. (2014). Neural bases of food perception: Coordinate-based meta-analyses of neuroimaging studies in multiple modalities. Obesity.

[29] Yeung A.W.K., Goto T.K., Leung W.K. (2018). Affective value, intensity and quality of liquid tastants/food discernment in the human brain: An activation likelihood estimation meta-analysis. Neuroimage.

[30] Chen E.Y., Zeffiro T.A. (2020). Hunger and BMI modulate neural responses to sweet stimuli: fMRI meta-analysis. Int. J. Obes..

[31] Sescousse G., Caldu X., Segura B., Dreher J.C. (2013). Processing of primary and secondary rewards: A quantitative meta-analysis and review of human functional neuroimaging studies. Neurosci. Biobehav. Rev..

[32] Pursey K.M., Stanwell P., Callister R.J., Brain K., Collins C.E., Burrows T.L. (2014). Neural responses to visual food cues according to weight status: A systematic review of functional magnetic resonance imaging studies. Front. Nutr..

[33] Eickhoff S.B., Laird A.R., Fox P.M., Lancaster J.L., Fox P.T. (2017). Implementation errors in the GingerALE Software: Description and recommendations. Hum. Brain Mapp..

[34] Cole T.J., Bellizzi M.C., Flegal K.M., Dietz W.H. (2000). Establishing a standard definition for child overweight and obesity worldwide: International survey. BMJ.

[35] Turkeltaub P.E., Eickhoff S.B., Laird A.R., Fox M., Wiener M., Fox P. (2012). Minimizing within-experiment and within-group effects in Activation Likelihood Estimation meta-analyses. Hum. Brain Mapp..

[36] Eickhoff S.B., Nichols T.E., Laird A.R., Hoffstaedter F., Amunts K., Fox P.T., Bzdok D., Eickhoff C.R. (2016). Behavior, sensitivity, and power of activation likelihood estimation characterized by massive empirical simulation. Neuroimage.

[37] Nichol A.D., Holle M.J., An R. (2018). Glycemic impact of non-nutritive sweeteners: A systematic review and meta-analysis of randomized controlled trials. Eur. J. Clin. Nutr..

[38] Basso F., Petit O., Le Bellu S., Lahlou S., Cancel A., Anton J.L. (2018). Taste at first (person) sight: Visual perspective modulates brain activity implicitly associated with viewing unhealthy but not healthy foods. Appetite.

[39] Basu T., Bao P., Lerner A., Anderson L., Page K., Stanczyk F., Mishell D., Segall-Gutierrez P. (2016). The Effect of Depo Medroxyprogesterone Acetate (DMPA) on Cerebral Food Motivation Centers: A Pilot Study using Functional Magnetic Resonance Imaging. Contraception.

[40] Beaver J.D., Lawrence A.D., van Ditzhuijzen J., Davis M.H., Woods A., Calder A.J. (2006). Individual differences in reward drive predict neural responses to images of food. J. Neurosci..

[41] Blechert J., Klackl J., Miedl S.F., Wilhelm F.H. (2016). To eat or not to eat: Effects of food availability on reward system activity during food picture viewing. Appetite.

[42] Carnell S., Benson L., Chang K.V., Wang Z., Huo Y., Geliebter A., Peterson B.S. (2017). Neural correlates of familial obesity risk and overweight in adolescence. Neuroimage.

[43] Chen P.A., Chavez R.S., Heatherton T.F. (2017). Structural integrity between executive control and reward regions of the brain predicts body fat percentage in chronic dieters. Cogn. Neurosci..

[44] Cornier M.A., McFadden K.L., Thomas E.A., Bechtell J.L., Eichman L.S., Bessesen D.H., Tregellas J.R. (2013). Differences in the neuronal response to food in obesity-resistant as compared to obesity-prone individuals. Physiol. Behav..

[45] Cornier M.A., Melanson E.L., Salzberg A.K., Bechtell J.L., Tregellas J.R. (2012). The effects of exercise on the neuronal response to food cues. Physiol. Behav..

[46] Cornier M.A., Salzberg A.K., Endly D.C., Bessesen D.H., Rojas D.C., Tregellas J.R. (2009). The effects of overfeeding on the neuronal response to visual food cues in thin and reduced-obese individuals. PLoS ONE.

[47] Cornier M.A., Von Kaenel S.S., Bessesen D.H., Tregellas J.R. (2007). Effects of overfeeding on the neuronal response to visual food cues. Am. J. Clin. Nutr..

[48] Davids S., Lauffer H., Thoms K., Jagdhuhn M., Hirschfeld H., Domin M., Hamm A., Lotze M. (2010). Increased dorsolateral prefrontal cortex activation in obese children during observation of food stimuli. Int. J. Obes..

[49] Doornweerd S., De Geus E.J., Barkhof F., Van Bloemendaal L., Boomsma D.I., Van Dongen J., Drent M.L., Willemsen G., Veltman D.J., IJzerman G.R. (2018). Brain reward responses to food stimuli among female monozygotic twins discordant for BMI. Brain Imaging Behav..

[50] English L.K., Fearnbach S.N., Wilson S.J., Fisher J.O., Savage J.S., Rolls B.J., Keller K.L. (2017). Food portion size and energy density evoke different patterns of brain activation in children. Am. J. Clin. Nutr..

[51] Evero N., Hackett L.C., Clark R.D., Phelan S., Hagobian T.A. (2012). Aerobic exercise reduces neuronal responses in food reward brain regions. J. Appl. Physiol..

[52] Frank S., Laharnar N., Kullmann S., Veit R., Canova C., Hegner Y.L., Fritsche A., Preissl H. (2010). Processing of food pictures: Influence of hunger, gender and calorie content. Brain Res..

[53] Frank S., Wilms B., Veit R., Ernst B., Thurnheer M., Kullmann S., Fritsche A., Birbaumer N., Preissl H., Schultes B. (2014). Altered brain activity in severely obese women may recover after Roux-en Y gastric bypass surgery. Int. J. Obes..

[54] Garcia-Garcia I., Kube J., Morys F., Schrimpf A., Kanaan A.S., Gaebler M., Villringer A., Dagher A., Horstmann A., Neumann J. (2020). Liking and left amygdala activity during food versus nonfood processing are modulated by emotional context. Cogn. Affect. Behav. Neurosci..

[55] Gearhardt A.N., Yokum S., Harris J.L., Epstein L.H., Lumeng J.C. (2020). Neural response to fast food commercials in adolescents predicts intake. Am. J. Clin. Nutr..

[56] Geliebter A., Pantazatos S.P., McOuatt H., Puma L., Gibson C.D., Atalayer D. (2013). Sex-based fMRI differences in obese humans in response to high vs. low energy food cues. Behav. Brain Res..

[57] Goldstone A.P., Prechtl de Hernandez C.G., Beaver J.D., Muhammed K., Croese C., Bell G., Durighel G., Hughes E., Waldman A.D., Frost G. (2009). Fasting biases brain reward systems towards high-calorie foods. Eur. J. Neurosci..

[58] Heni M., Kullmann S., Ketterer C., Guthoff M., Bayer M., Staiger H., Machicao F., Haring H.U., Preissl H., Veit R. (2014). Differential effect of glucose ingestion on the neural processing of food stimuli in lean and overweight adults. Hum. Brain Mapp..

[59] Hermann P., Gal V., Kobor I., Kirwan C.B., Kovacs P., Kitka T., Lengyel Z., Balint E., Varga B., Cseko C. (2019). Efficacy of weight loss intervention can be predicted based on early alterations of fMRI food cue reactivity in the striatum. Neuroimage Clin..

[60] Horster I., Nickel K., Holovics L., Schmidt S., Endres D., Tebartz van Elst L., Zeeck A., Maier S., Joos A. (2020). A Neglected Topic in Neuroscience: Replicability of fMRI Results with Specific Reference to ANOREXIA NERVOSA. Front. Psychiatry.

[61] Jastreboff A.M., Lacadie C., Seo D., Kubat J., Van Name M.A., Giannini C., Savoye M., Constable R.T., Sherwin R.S., Caprio S. (2014). Leptin is associated with exaggerated brain reward and emotion responses to food images in adolescent obesity. Diabetes Care.

[62] Jastreboff A.M., Sinha R., Lacadie C., Small D.M., Sherwin R.S., Potenza M.N. (2013). Neural correlates of stress- and food cue-induced food craving in obesity: Association with insulin levels. Diabetes Care.

[63] Jensen C.D., Kirwan C.B. (2015). Functional brain response to food images in successful adolescent weight losers compared with normal-weight and overweight controls. Obesity.

[64] Karra E., O’Daly O.G., Choudhury A.I., Yousseif A., Millership S., Neary M.T., Scott W.R., Chandarana K., Manning S., Hess M.E. (2013). A link between FTO, ghrelin, and impaired brain food-cue responsivity. J. Clin. Investig..

[65] Killgore W.D., Yurgelun-Todd D.A. (2005). Developmental changes in the functional brain responses of adolescents to images of high and low-calorie foods. Dev. Psychobiol..

[66] Killgore W.D.S., Young A.D., Femia L.A., Bogorodzki P., Rogowska J., Yurgelun-Todd D.A. (2003). Cortical and limbic activation during viewing of high- versus low-calorie foods. NeuroImage.

[67] Kim K.R., Ku J., Lee J.H., Lee H., Jung Y.C. (2012). Functional and effective connectivity of anterior insula in anorexia nervosa and bulimia nervosa. Neurosci. Lett..

[68] Le T.M., Zhornitsky S., Wang W., Zhang S., Li C.R. (2021). Problem drinking alters gray matter volume and food cue responses of the lateral orbitofrontal cortex. Addict. Biol..

[69] Li G., Hu Y., Zhang W., Ding Y., Wang Y., Wang J., He Y., Lv G., von Deneen K.M., Zhao Y. (2021). Resting activity of the hippocampus and amygdala in obese individuals predicts their response to food cues. Addict. Biol..

[70] Luo S., Alves J., Hardy K., Wang X., Monterosso J., Xiang A.H., Page K.A. (2019). Neural processing of food cues in pre-pubertal children. Pediatr. Obes..

[71] Luo S., Romero A., Adam T.C., Hu H.H., Monterosso J., Page K.A. (2013). Abdominal fat is associated with a greater brain reward response to high-calorie food cues in Hispanic women. Obesity.

[72] Malik S., McGlone F., Dagher A. (2011). State of expectancy modulates the neural response to visual food stimuli in humans. Appetite.

[73] Masterson T.D., Kirwan C.B., Davidson L.E., LeCheminant J.D. (2016). Neural reactivity to visual food stimuli is reduced in some areas of the brain during evening hours compared to morning hours: An fMRI study in women. Brain Imaging Behav..

[74] Mengotti P., Foroni F., Rumiati R.I. (2019). Neural correlates of the energetic value of food during visual processing and response inhibition. Neuroimage.

[75] Merchant J.S., Cosme D., Giuliani N.R., Dirks B., Berkman E.T. (2020). Neural Substrates of Food Valuation and Its Relationship with BMI and Healthy Eating in Higher BMI Individuals. Front. Behav. Neurosci..

[76] Murdaugh D.L., Cox J.E., Cook E.W., Weller R.E. (2012). fMRI reactivity to high-calorie food pictures predicts short- and long-term outcome in a weight-loss program. Neuroimage.

[77] Murray E., Brouwer S., McCutcheon R., Harmer C.J., Cowen P.J., McCabe C. (2014). Opposing neural effects of naltrexone on food reward and aversion: Implications for the treatment of obesity. Psychopharmacology.

[78] Neseliler S., Tannenbaum B., Zacchia M., Larcher K., Coulter K., Lamarche M., Marliss E.B., Pruessner J., Dagher A. (2017). Academic stress and personality interact to increase the neural response to high-calorie food cues. Appetite.

[79] Nummenmaa L., Hirvonen J., Hannukainen J.C., Immonen H., Lindroos M.M., Salminen P., Nuutila P. (2012). Dorsal striatum and its limbic connectivity mediate abnormal anticipatory reward processing in obesity. PLoS ONE.

[80] Passamonti L., Rowe J.B., Schwarzbauer C., Ewbank M.P., von dem Hagen E., Calder A.J. (2009). Personality predicts the brain’s response to viewing appetizing foods: The neural basis of a risk factor for overeating. J. Neurosci..

[81] Pursey K.M., Contreras-Rodriguez O., Collins C.E., Stanwell P., Burrows T.L. (2019). Food Addiction Symptoms and Amygdala Response in Fasted and Fed States. Nutrients.

[82] Rapuano K.M., Huckins J.F., Sargent J.D., Heatherton T.F., Kelley W.M. (2016). Individual Differences in Reward and Somatosensory-Motor Brain Regions Correlate with Adiposity in Adolescents. Cereb. Cortex.

[83] Rothemund Y., Preuschhof C., Bohner G., Bauknecht H.C., Klingebiel R., Flor H., Klapp B.F. (2007). Differential activation of the dorsal striatum by high-calorie visual food stimuli in obese individuals. Neuroimage.

[84] Santel S., Baving L., Krauel K., Munte T.F., Rotte M. (2006). Hunger and satiety in anorexia nervosa: fMRI during cognitive processing of food pictures. Brain Res..

[85] Schienle A., Schafer A., Hermann A., Vaitl D. (2009). Binge-eating disorder: Reward sensitivity and brain activation to images of food. Biol. Psychiatry.

[86] Simmons W.K., Martin A., Barsalou L.W. (2005). Pictures of appetizing foods activate gustatory cortices for taste and reward. Cereb. Cortex.

[87] Smeets P.A., Kroese F.M., Evers C., de Ridder D.T. (2013). Allured or alarmed: Counteractive control responses to food temptations in the brain. Behav. Brain Res..

[88] St-Onge M.P., Wolfe S., Sy M., Shechter A., Hirsch J. (2014). Sleep restriction increases the neuronal response to unhealthy food in normal-weight individuals. Int. J. Obes..

[89] Van Bloemendaal L., RG I.J., Ten Kulve J.S., Barkhof F., Konrad R.J., Drent M.L., Veltman D.J., Diamant M. (2014). GLP-1 receptor activation modulates appetite- and reward-related brain areas in humans. Diabetes.

[90] Van Meer F., van der Laan L.N., Charbonnier L., Viergever M.A., Adan R.A., Smeets P.A., Consortium I.F. (2016). Developmental differences in the brain response to unhealthy food cues: An fMRI study of children and adults. Am. J. Clin. Nutr..

[91] Wabnegger A., Schwab D., Schienle A. (2018). Aversive aftertaste changes visual food cue reactivity: An fMRI study on cross-modal perception. Neurosci. Lett..

[92] Wagner D.D., Boswell R.G., Kelley W.M., Heatherton T.F. (2012). Inducing negative affect increases the reward value of appetizing foods in dieters. J. Cogn. Neurosci..

[93] Wang Y., Dong D., Todd J., Du J., Yang Z., Lu H., Chen H. (2016). Neural correlates of restrained eaters’ high susceptibility to food cues: An fMRI study. Neurosci. Lett..

[94] Yokum S., Bohon C., Berkman E., Stice E. (2021). Test-retest reliability of functional MRI food receipt, anticipated receipt, and picture tasks. Am. J. Clin. Nutr..

[95] Van Meer A.F. (2017). Neural Processing of Healthy Foods in Normal-Weight and Overweight Children and Adults. Ph.D. Thesis.

[96] Yang Y., Morys F., Li J., Wu Q., Chen H. (2021). Food-Specific Go/No-Go Training for Overweight Individuals: Brain Imaging Data Suggest Inhibition Shapes Food Evaluation. Soc. Cogn. Affect. Neurosci. Unpublished manuscript.

[97] Van den Akker K., Stewart K., Antoniou E.E., Palmberg A., Jansen A. (2014). Food Cue Reactivity, Obesity, and Impulsivity: Are They Associated?. Curr. Addict. Rep..

[98] Devoto F., Zapparoli L., Bonandrini R., Berlingeri M., Ferrulli A., Luzi L., Banfi G., Paulesu E. (2018). Hungry brains: A meta-analytical review of brain activation imaging studies on food perception and appetite in obese individuals. Neurosci. Biobehav. Rev..

[99] LeDoux J.E. (2000). Emotion circuits in the brain. Annu. Rev. Neurosci..

[100] Janak P.H., Tye K.M. (2015). From circuits to behaviour in the amygdala. Nature.

[101] Zheng J., Anderson K.L., Leal S.L., Shestyuk A., Gulsen G., Mnatsakanyan L., Vadera S., Hsu F.P., Yassa M.A., Knight R.T. (2017). Amygdala-hippocampal dynamics during salient information processing. Nat. Commun..

[102] Richter-Levin G., Akirav I. (2003). Emotional tagging of memory formation--in the search for neural mechanisms. Brain Res. Brain Res. Rev..

[103] Rudebeck P.H., Mitz A.R., Chacko R.V., Murray E.A. (2013). Effects of amygdala lesions on reward-value coding in orbital and medial prefrontal cortex. Neuron.

[104] Liu X., Hairston J., Schrier M., Fan J. (2011). Common and distinct networks underlying reward valence and processing stages: A meta-analysis of functional neuroimaging studies. Neurosci. Biobehav. Rev..

[105] Hill-Bowen L.D., Riedel M.C., Poudel R., Salo T., Flannery J.S., Camilleri J.A., Eickhoff S.B., Laird A.R., Sutherland M.T. (2021). The cue-reactivity paradigm: An ensemble of networks driving attention and cognition when viewing drug and natural reward-related stimuli. Neurosci. Biobehav. Rev..

[106] Kringelbach M.L., O’Doherty J., Rolls E.T., Andrews C. (2003). Activation of the human orbitofrontal cortex to a liquid food stimulus is correlated with its subjective pleasantness. Cereb. Cortex.

[107] Simmons W.K., Rapuano K.M., Ingeholm J.E., Avery J., Kallman S., Hall K.D., Martin A. (2014). The ventral pallidum and orbitofrontal cortex support food pleasantness inferences. Brain Struct. Funct..

[108] Londeree A.M., Wagner D.D. (2021). The orbitofrontal cortex spontaneously encodes food health and contains more distinct representations for foods highest in tastiness. Soc. Cogn. Affect. Neurosci..

[109] Rolls E.T. (2016). Functions of the anterior insula in taste, autonomic, and related functions. Brain Cogn..

[110] Dagher A. (2012). Functional brain imaging of appetite. Trends Endocrinol. Metab..

[111] Naqvi N.H., Bechara A. (2009). The hidden island of addiction: The insula. Trends Neurosci..

[112] Pelchat M.L., Johnson A., Chan R., Valdez J., Ragland J.D. (2004). Images of desire: Food-craving activation during fMRI. Neuroimage.

[113] Zhu J.N., Wang J.J. (2008). The cerebellum in feeding control: Possible function and mechanism. Cell. Mol. Neurobiol..

[114] Caligiore D., Arbib M.A., Miall R.C., Baldassarre G. (2019). The super-learning hypothesis: Integrating learning processes across cortex, cerebellum and basal ganglia. Neurosci. Biobehav. Rev..

[115] Hanlon C.A., Dowdle L.T., Naselaris T., Canterberry M., Cortese B.M. (2014). Visual cortex activation to drug cues: A meta-analysis of functional neuroimaging papers in addiction and substance abuse literature. Drug Alcohol Depend..

[116] Ko C.H., Liu G.C., Yen J.Y., Chen C.Y., Yen C.F., Chen C.S. (2013). Brain correlates of craving for online gaming under cue exposure in subjects with Internet gaming addiction and in remitted subjects. Addict. Biol..

[117] Drewnowski A. (1997). Taste preferences and food intake. Annu. Rev. Nutr..

[118] Morys F., Garcia-Garcia I., Dagher A. (2020). Is obesity related to enhanced neural reactivity to visual food cues? A review and meta-analysis. Soc. Cogn. Affect. Neurosci..

[119] Meng X., Huang D., Ao H., Wang X., Gao X. (2020). Food cue recruits increased reward processing and decreased inhibitory control processing in the obese/overweight: An activation likelihood estimation meta-analysis of fMRI studies. Obes. Res. Clin. Pract..

[120] Stice E., Yokum S. (2016). Neural vulnerability factors that increase risk for future weight gain. Psychol. Bull..

