# Supplementary material for: Brain Responses to High-Calorie Visual Food Cues in Individuals with Normal-Weight or Obesity: An Activation Likelihood Estimation Meta-Analysis
Source: Brain Sci. 2021 Nov 30;11(12):1587. doi: 10.3390/brainsci11121587 (PMC8699077; doi:10.3390/brainsci11121587)
Supplement: Supplementary file 1 [file brainsci-11-01587-s001.zip › brainsci-1469168-supplementary.pdf]

## Supplementary materials

1. Table S1. PRISMA Checklist

2. Table S2 Quality assessment of each included study

3. Table S3. Overall Activation Likelihood Estimation meta-analysis of high-calorie visual food stimuli relative to the non-food condition using 43 independent samples.

4. Table S4. Conjunction and contrast analyses on activations of high-calorie visual food stimuli relative to the non-food condition between obesity and normal-weight.

5. Table S5. Overall Activation Likelihood Estimation meta-analysis of high-calorie visual food stimuli relative to the low-calorie visual food condition using 39 independent samples.

6. Table S6. Conjunction and contrast analyses on activations of high-calorie visual food stimuli relative to the low-calorie visual food condition between obesity and normal-weight.

**Table S1. PRISMA Checklist*.**

| Section/topic | # | Checklist item | Reported on page # |
|---|---|---|---|
| **TITLE** | | | |
| Title | 1 | Identify the report as a systematic review, meta-analysis, or both. | 1 |
| **ABSTRACT** | | | |
| Structured summary | 2 | Provide a structured summary including, as applicable: background; objectives; data sources; study eligibility criteria, participants, and interventions; study appraisal and synthesis methods; results; limitations; conclusions and implications of key findings; systematic review registration number. | 2 |
| **INTRODUCTION** | | | |
| Rationale | 3 | Describe the rationale for the review in the context of what is already known. | 4 |
| Objectives | 4 | Provide an explicit statement of questions being addressed with reference to participants, interventions, comparisons, outcomes, and study design (PICOS). | |
| **METHODS** | | | |
| Protocol and registration | 5 | Indicate if a review protocol exists, if and where it can be accessed (e.g., Web address), and, if available, provide registration information including registration number. | 5 |
| Eligibility criteria | 6 | Specify study characteristics (e.g., PICOS, length of follow-up) and report characteristics (e.g., years considered, language, publication status) used as criteria for eligibility, giving rationale. | 6 |
| Information sources | 7 | Describe all information sources (e.g., databases with dates of coverage, contact with study authors to identify additional studies) in the search and date last searched. | 6 |
| Search | 8 | Present full electronic search strategy for at least one database, including any limits used, such that it could be repeated. | 5 |
| Study selection | 9 | State the process for selecting studies (i.e., screening, eligibility, included in systematic review, and, if applicable, included in the meta-analysis). | 6 |
| Data collection process | 10 | Describe method of data extraction from reports (e.g., piloted forms, independently, in duplicate) and any processes for obtaining and confirming data from investigators. | 6，7 |
| Data items | 11 | List and define all variables for which data were sought (e.g., PICOS, funding sources) and any assumptions and simplifications made. | 9-15 |
| Risk of bias in individual studies | 12 | Describe methods used for assessing risk of bias of individual studies (including specification of whether this was done at the study or outcome level), and how this information is to be used in any data synthesis. | S4-5 |

| | | | |
|---|---|---|---|
| Summary measures | 13 | State the principal summary measures (e.g., risk ratio, difference in means). | 7 |
| Synthesis of results | 14 | Describe the methods of handling data and combining results of studies, if done, including measures of consistency (e.g., $I^2$) for each meta-analysis. | 7 |
| Risk of bias across studies | 15 | Specify any assessment of risk of bias that may affect the cumulative evidence (e.g., publication bias, selective reporting within studies). | n.a |
| Additional analyses | 16 | Describe methods of additional analyses (e.g., sensitivity or subgroup analyses, meta-regression), if done, indicating which were pre-specified. | 8 |
| **RESULTS** | | | |
| Study selection | 17 | Give numbers of studies screened, assessed for eligibility, and included in the review, with reasons for exclusions at each stage, ideally with a flow diagram. | 8 |
| Study characteristics | 18 | For each study, present characteristics for which data were extracted (e.g., study size, PICOS, follow-up period) and provide the citations. | 9-15 |
| Risk of bias within studies | 19 | Present data on risk of bias of each study and, if available, any outcome level assessment (see item 12). | S4-5 |
| Results of individual studies | 20 | For all outcomes considered (benefits or harms), present, for each study: (a) simple summary data for each intervention group (b) effect estimates and confidence intervals, ideally with a forest plot. | n.a |
| Synthesis of results | 21 | Present results of each meta-analysis done, including confidence intervals and measures of consistency. | 16-21 |
| Risk of bias across studies | 22 | Present results of any assessment of risk of bias across studies (see Item 15). | |
| Additional analysis | 23 | Give results of additional analyses, if done (e.g., sensitivity or subgroup analyses, meta-regression [see Item 16]). | n.a |
| **DISCUSSION** | | | |
| Summary of evidence | 24 | Summarize the main findings including the strength of evidence for each main outcome; consider their relevance to key groups (e.g., healthcare providers, users, and policy makers). | 22 |
| Limitations | 25 | Discuss limitations at study and outcome level (e.g., risk of bias), and at review-level (e.g., incomplete retrieval of identified research, reporting bias). | 26 |
| Conclusions | 26 | Provide a general interpretation of the results in the context of other evidence, and implications for future research. | 26-27 |
| **FUNDING** | | | |
| Funding | 27 | Describe sources of funding for the systematic review and other support (e.g., supply of data); role of funders for the systematic review. | 42 |

*Note: Moher D, Liberati A, Tetzlaff J, Altman DG, The PRISMA Group (2009). Preferred Reporting Items for Systematic Reviews and Meta-Analyses: The PRISMA Statement. PLoS Med 6(7): e1000097. doi:10.1371/journal.pmed1000097. For more information, visit www.prisma-statement.org.

Abbreviations: s, supplementary material. n.a = not applicable.

Table S2 Quality assessment of each included study

| Study | Criterion | | | | | | | Total Score |
| --- | --- | --- | --- | --- | --- | --- | --- | --- |
| | 1 | 2 | 3 | 4 | 5 | 6 | 7 | |
| Basso et al., 2018 | 2 | 2 | 2 | 0 | 2 | 2 | 2 | 12 |
| Basu et al., 2016 | 2 | 2 | 2 | 0 | 0 | 2 | 0 | 8 |
| Beaver et al., 2006 | 2 | 2 | 2 | 0 | 2 | 2 | 0 | 10 |
| Blechert et al., 2016 | 2 | 2 | 2 | 0 | 2 | 2 | 2 | 12 |
| Carnell et al., 2017 | 2 | 2 | 2 | 0 | 2 | 2 | 0 | 10 |
| Chen et al., 2017 | 2 | 2 | 2 | 0 | 2 | 2 | 2 | 12 |
| Cornier et al., 2007 | 2 | 2 | 2 | 0 | 2 | 2 | 2 | 12 |
| Cornier et al., 2009 | 2 | 2 | 2 | 0 | 2 | 2 | 2 | 12 |
| Cornier et al., 2012 | 2 | 2 | 2 | 0 | 2 | 2 | 0 | 10 |
| Cornier et al., 2013 | 2 | 2 | 2 | 0 | 2 | 2 | 2 | 12 |
| Davids et al., 2010 | 2 | 2 | 2 | 0 | 2 | 2 | 2 | 12 |
| Doornweerd et al., 2018 | 2 | 2 | 2 | 0 | 2 | 2 | 2 | 12 |
| English et al., 2017 | 2 | 2 | 2 | 0 | 2 | 2 | 2 | 12 |
| Evero et al., 2012 | 2 | 2 | 2 | 0 | 2 | 2 | 2 | 12 |
| Frank et al., 2010 | 2 | 2 | 2 | 0 | 2 | 2 | 0 | 10 |
| Frank et al., 2014 | 2 | 2 | 2 | 0 | 2 | 2 | 2 | 12 |
| García-García et al., 2020 | 2 | 2 | 2 | 0 | 2 | 2 | 2 | 12 |
| Gearhardt et al., 2020 | 2 | 2 | 2 | 0 | 2 | 2 | 2 | 12 |
| Geliebter et al., 2013 | 2 | 2 | 2 | 0 | 2 | 2 | 2 | 12 |
| Goldstone et al., 2009 | 2 | 2 | 2 | 0 | 2 | 2 | 2 | 12 |
| Heni et al., 2014 | 2 | 2 | 2 | 0 | 2 | 2 | 2 | 12 |
| Hermann et al., 2019 | 2 | 2 | 2 | 0 | 2 | 2 | 2 | 12 |
| Horster et al., 2020 | 2 | 2 | 2 | 0 | 2 | 2 | 2 | 12 |
| Jastreboff et al., 2013 | 2 | 2 | 2 | 0 | 2 | 2 | 2 | 12 |
| Jastreboff et al., 2014 | 2 | 2 | 2 | 0 | 2 | 2 | 1 | 11 |
| Jensen & Kirwan, 2015 | 2 | 2 | 2 | 0 | 2 | 2 | 2 | 12 |
| Karra et al., 2013 | 2 | 2 | 2 | 0 | 2 | 2 | 2 | 12 |
| Killgore et al., 2003 | 2 | 2 | 2 | 0 | 2 | 2 | 0 | 10 |
| Killgore et al., 2006 | 2 | 2 | 2 | 0 | 2 | 2 | 0 | 10 |

| | | | | | | | | |
|---|---|---|---|---|---|---|---|---|
| Kim et al., 2012 | 2 | 2 | 2 | 0 | 2 | 2 | 2 | 12 |
| Le et al., 2021 | 2 | 2 | 2 | 0 | 2 | 2 | 2 | 12 |
| Li et al., 2021 | 2 | 2 | 2 | 0 | 2 | 2 | 2 | 12 |
| Luo et al., 2013 | 2 | 2 | 2 | 0 | 2 | 2 | 0 | 10 |
| Luo et al., 2019 | 2 | 2 | 2 | 0 | 2 | 2 | 2 | 12 |
| Malik et al., 2011 | 2 | 2 | 2 | 0 | 2 | 2 | 0 | 10 |
| Masterson et al., 2016 | 2 | 2 | 2 | 0 | 2 | 2 | 0 | 10 |
| Mengotti et al., 2019 | 2 | 2 | 2 | 0 | 2 | 2 | 2 | 12 |
| Merchant et al., 2020 | 2 | 2 | 2 | 0 | 2 | 2 | 2 | 12 |
| Murdaugh et al., 2012 | 2 | 2 | 2 | 0 | 2 | 2 | 1 | 11 |
| Murray et al., 2014 | 2 | 2 | 2 | 0 | 2 | 2 | 2 | 12 |
| Neseliler et al., 2017 | 2 | 2 | 2 | 0 | 2 | 2 | 2 | 12 |
| Nummenmaa et al., 2012 | 2 | 2 | 2 | 0 | 2 | 2 | 2 | 12 |
| Passamonti et al., 2009 | 2 | 2 | 2 | 0 | 2 | 2 | 2 | 12 |
| Pursey et al., 2019 | 2 | 2 | 2 | 0 | 2 | 2 | 0 | 10 |
| Rapuano et al., 2016 | 2 | 2 | 2 | 0 | 2 | 2 | 2 | 12 |
| Rothemund et al., 2007 | 2 | 2 | 2 | 0 | 2 | 2 | 0 | 10 |
| Santel et al., 2006 | 2 | 2 | 2 | 0 | 2 | 2 | 0 | 10 |
| Schienle et al., 2009 | 2 | 2 | 2 | 0 | 2 | 2 | 0 | 10 |
| Simmons et al., 2005 | 2 | 2 | 2 | 0 | 2 | 2 | 0 | 10 |
| Smeets et al., 2013 | 2 | 2 | 2 | 0 | 2 | 2 | 2 | 12 |
| St-Onge et al., 2014 | 2 | 2 | 2 | 0 | 2 | 2 | 2 | 12 |
| van Bloemendaal et al., 2014 | 2 | 2 | 2 | 0 | 2 | 2 | 2 | 12 |
| van Meer et al., 2016 | 2 | 2 | 2 | 0 | 2 | 2 | 2 | 12 |
| van Meer, 2017 | 2 | 2 | 2 | 0 | 2 | 2 | 2 | 12 |
| Wabnegger et al., 2018 | 2 | 2 | 2 | 0 | 2 | 2 | 2 | 12 |
| Wagner et al., 2012 | 2 | 2 | 2 | 0 | 2 | 2 | 2 | 12 |
| Wang et al., 2016 | 2 | 2 | 2 | 0 | 2 | 2 | 2 | 12 |
| Yang et al., 2021 | 2 | 2 | 2 | 0 | 2 | 2 | 2 | 12 |
| Yokum et al., 2021 | 2 | 2 | 2 | 0 | 2 | 2 | 2 | 12 |

Scores for each criterion range from 0 to 2, with 0 being not reported or not met, 1 being partially met, and 2 being completely met. Thus, the total score ranges from 0 to 14. The criteria were as

follows: (1) Was the research question clearly stated? (2) Were the inclusion and exclusion criteria clearly stated? (3) Were study participants' BMIs clearly reported? (4) Was a power analysis conducted to calculate the required sample size? (5) Was the dropout rate or data exclusion rate 20% or lower? (6) Was the population referenced in the conclusion appropriate? (7) Were there 20 or more study participants?

**Table S3.** Overall Activation Likelihood Estimation meta-analysis of high-calorie visual food stimuli relative to the non-food condition using 43 independent samples.

| **Cluster** | **Cluster size ($mm^3$)** | Brain region | Peak voxel MNI coordinates | | | ALE value ($\times 10^{-2}$) | Z | Contributing samples | |
|---|---|---|---|---|---|---|---|---|---|
| | | | X | Y | Z | | | No. | % |
| 1 | 3176 | L Orbitofrontal Cortex | -26 | 34 | -14 | 6.24 | 8.65 | 15 | 35% |
| 2 | 2912 | L Insula | -38 | -6 | 6 | 6.89 | 9.25 | 17 | 40% |
| 3 | 2688 | R Lingual Gyrus | 18 | -94 | 4 | 2.63 | 4.77 | 14 | 33% |
| 4 | 2320 | L Lingual Gyrus | -12 | -94 | -8 | 2.61 | 4.74 | 11 | 26% |
| 5 | 2136 | L Fusiform Gyrus | -46 | -68 | -8 | 3.73 | 6.08 | 12 | 28% |
| 6 | 2088 | R Fusiform Gyrus | 46 | -66 | -10 | 2.79 | 3.35 | 11 | 26% |
| 7 | 2048 | L Amygdala | -22 | -2 | -20 | 2.84 | 5.03 | 11 | 26% |
| 8 | 1672 | R Insula | 38 | 8 | -12 | 6.00 | 8.41 | 10 | 23% |
| 9 | 1440 | R Insula | 40 | -4 | 4 | 4.59 | 7.01 | 9 | 21% |
| 10 | 1296 | R Superior Parietal Lobule | 30 | -56 | 54 | 4.25 | 6.65 | 7 | 16% |
| 11 | 1144 | L Inferior Frontal Gyrus | -36 | 6 | -16 | 3.04 | 5.28 | 7 | 16% |
| 12 | 1144 | R Orbitofrontal Cortex | 24 | 30 | -16 | 3.42 | 5.73 | 7 | 16% |
| 13 | 864 | R Amygdala | 20 | -2 | -18 | 3.13 | 5.38 | 6 | 14% |
| 14 | 840 | R Precuneus | 28 | -72 | 34 | 2.99 | 5.22 | 5 | 12% |

Note: L: left, R: right. These presented clusters were at $p < 0.001$ corrected for multiple comparisons using cluster-level family-wise error correction at $p < 0.01$ (1,000 permutations)

**Table S4.** Conjunction and contrast analyses on activations of high-calorie visual food stimuli relative to the non-food condition between obesity and normal-weight

| Cluster | Cluster size ($mm^3$) | Brain region | Peak voxel MNI coordinates | | | ALE value ($\times 10^{-2}$) / Z |
|---|---|---|---|---|---|---|
| | | | X | Y | Z | |
| Obesity ∩ Normal-weight | 936 | L Orbitofrontal Cortex | -26 | 34 | -16 | 2.48 |
| Obesity > Normal-weight | None | | | | | |
| Obesity < Normal-weight | None | | | | | |

Note: L: left, R: right. These presented clusters were at $p < .01$ with 10,00 permutations and a minimum cluster size of 200 $mm^3$.

**Table S5.** Overall Activation Likelihood Estimation meta-analysis of high-calorie visual food stimuli relative to the low-calorie visual food condition using 39 independent samples.

| **Cluster** | **Cluster size ($mm^3$)** | Brain region | Peak voxel MNI coordinates | | | ALE value ($\times 10^{-2}$) | Z | Contributing samples | |
|---|---|---|---|---|---|---|---|---|---|
| | | | X | Y | Z | | | No. | % |
| 1 | 1880 | R Middle Occipital Gyrus | 36 | -84 | 12 | 3.56 | 6.06 | 8 | 21% |
| 2 | 1432 | R Orbitofrontal Cortex | 30 | 30 | -12 | 6.89 | 9.25 | 8 | 21% |
| 3 | 1256 | R Fusiform Gyrus | 50 | -60 | -10 | 2.63 | 4.77 | 7 | 18% |
| 4 | 1192 | R Culmen | 28 | -46 | -16 | 2.61 | 4.74 | 5 | 13% |
| 5 | 1120 | L Lingual Gyrus | -16 | -100 | -6 | 3.73 | 6.08 | 5 | 13% |

Note: L: left, R: right. These presented clusters were at $p < 0.001$ corrected for multiple comparisons using cluster-level family-wise error correction at $p < 0.01$ (1,000 permutations)

**Table S6.** Conjunction and contrast analyses on activations of high-calorie visual food stimuli relative to the low-calorie visual food condition between obesity and normal-weight

| Cluster | Cluster size ($mm^3$) | Brain region | Peak voxel MNI coordinates | | | ALE value ($\times 10^{-2}$) / Z |
|---|---|---|---|---|---|---|
| | | | X | Y | Z | |
| Obesity ∩ Normal-weight | None | | | | | |
| Obesity > Normal-weight | None | | | | | |
| Obesity < Normal-weight | None | | | | | |

Note: L: left, R: right. These presented clusters were at $p < .01$ with 10,00 permutations and a minimum cluster size of 200 $mm^3$.
